# Vitamin D Deficiency in the Pregnant Population at University Hospital Birmingham: Does It Justify Universal Screening?

**DOI:** 10.7759/cureus.100847

**Published:** 2026-01-05

**Authors:** Shaista Zubair, Umme Habiba, Maimoona Iqbal, Shalini Patni

**Affiliations:** 1 Department of Obstetrics and Gynaecology, University Hospital Birmingham, Birmingham, GBR

**Keywords:** 25-hydroxyvitamin d, antenatal screening, maternal health, pregnancy, vitamin d deficiency

## Abstract

Background: Vitamin D deficiency is a growing global health concern, particularly among pregnant women, due to its associations with adverse maternal and neonatal outcomes. Birmingham represents a unique high-risk population with substantial ethnic diversity and socioeconomic deprivation, both of which may predispose to low vitamin D levels. This study aimed to determine the prevalence of vitamin D deficiency among pregnant women attending antenatal care at a large tertiary hospital and to evaluate whether risk-based screening adequately identifies deficient individuals.

Methods: This retrospective observational study included pregnant women who underwent serum 25-hydroxyvitamin D (25(OH)D) testing at University Hospital Birmingham between January and December 2022. A total of 2,757 records were reviewed to determine the overall prevalence. A randomly selected cohort of 300 women (100 normal, 100 mildly deficient, and 100 severely deficient) with complete clinical data was used to evaluate demographic and clinical risk factors. Vitamin D status was categorized according to the UK Public Health/National Institute for Health and Care Excellence (NICE) criteria. Statistical analyses included one-sample t-tests and chi-squared tests, with p < 0.05 considered significant.

Results: Among 2,757 women tested, 1,701 (61.7%) had insufficient or deficient vitamin D levels, significantly exceeding the 60% threshold (p = 0.034). Severe deficiency (<25 nmol/L) was present in 634 women (23%), also significantly higher than the hypothesized 21% prevalence (p = 0.006). In the 300-patient comparison cohort, the mean age was 29.94 ± 6.73 years, and the mean body mass index (BMI) was 29.33 ± 7.72 kg/m². Distribution of age, BMI, ethnicity, deprivation index, and comorbidities did not differ significantly across vitamin D categories, with all p-values > 0.05. No traditional risk factor showed a significant association with vitamin D status. Parity was the only variable significantly associated with vitamin D status (p = 0.002).

Conclusion: Vitamin D deficiency is highly prevalent among pregnant women in this ethnically diverse Birmingham population. Most classical risk factors did not reliably predict deficiency, although multiparity emerged as a significant contributor. Given the high burden of deficiency, low cost of testing, and limited discriminatory value of existing risk-based criteria, universal vitamin D screening at booking may be justified. Further prospective studies are needed to evaluate the impact of universal testing and targeted supplementation on maternal and neonatal outcomes.

## Introduction

Vitamin D is a fat-soluble secosteroid hormone involved not only in calcium and phosphate homeostasis but also in a wide range of metabolic, endocrine, and immunologic processes essential to pregnancy [1]. During gestation, adequate maternal vitamin D is required for optimal placental implantation, modulation of inflammatory pathways, glucose metabolism, and fetal skeletal mineralization [2]. Vitamin D receptors are expressed in the placenta, decidua, uterus, and multiple fetal tissues, highlighting its physiologic influence beyond bone health [3]. Despite its broad biologic significance, vitamin D deficiency remains a global public health concern, and pregnant women represent a particularly vulnerable group due to increased metabolic demands, limited dietary sources, and seasonal or geographic constraints on sunlight exposure [4].

The classification of vitamin D deficiency, however, remains inconsistent across major international bodies, posing challenges for both research interpretation and clinical management [5]. The Endocrine Society defines serum 25-hydroxyvitamin D (25(OH)D) concentrations < 20 ng/mL as deficient and 21-29 ng/mL as insufficient, whereas the Institute of Medicine considers concentrations > 20 ng/mL sufficient for most individuals [6]. Additional pediatric, bone health, and endocrine societies provide alternative thresholds, and emerging evidence suggests that higher concentrations may be necessary during pregnancy to optimize perinatal outcomes [7]. In the United Kingdom, vitamin D status is commonly categorized as sufficient (>50 nmol/L), insufficient (25-49.9 nmol/L), and deficient (<25 nmol/L), consistent with National Institute for Health and Care Excellence (NICE) guidelines [8]. These variations underscore the ongoing debate regarding optimal vitamin D targets in pregnancy and emphasize the need for population-specific data.

Vitamin D deficiency in pregnancy is widely recognized across the world, with prevalence estimates exceeding 50%-80% in some regions [9]. Risk is disproportionately higher among women with darker skin pigmentation, obesity, limited sunlight exposure, malabsorption syndromes, and those wearing culturally modest clothing. Socioeconomic deprivation and poor nutritional intake further compound risk [10]. Within the United Kingdom, deficiency is particularly common among South Asian, Black, and Middle Eastern women-groups that constitute a large proportion of the population in cities such as Birmingham [11]. This raises concern for pregnant populations living in northern latitudes, where endogenous vitamin D synthesis is limited for nearly half the year [12].

Low maternal vitamin D levels have been associated with several adverse obstetric and neonatal outcomes, including pre-eclampsia, gestational diabetes mellitus, preterm birth, small-for-gestational-age infants, and impaired fetal bone mineralization [13]. Deficiency has also been linked to increased rates of cesarean delivery, neonatal hypocalcemic seizures, and altered long-term health outcomes in offspring, such as respiratory morbidity and metabolic dysregulation [14]. These associations, although not universally causal, highlight the potential clinical consequences of unrecognized or undertreated deficiency during pregnancy.

Despite its clinical relevance, screening practices remain variable. The United Kingdom currently adopts a risk-based screening strategy, recommending vitamin D testing only for women with specific risk factors. However, local audits and perinatal mortality review themes at University Hospital Birmingham (UHB) have identified frequent missed opportunities to diagnose and treat deficiency, suggesting that risk-based approaches may not adequately capture the burden of disease in ethnically diverse and socioeconomically deprived populations. Limited local prevalence data further hinder the development of effective and consistent screening guidelines. Therefore, this study aimed to determine the incidence and severity of vitamin D deficiency among pregnant women within our catchment population and to evaluate whether our findings support the need for universal vitamin D screening at booking in this high-risk region.

## Materials and methods

Study design and setting

This retrospective observational study was conducted at UHB, a large tertiary care center serving an ethnically diverse and socioeconomically deprived population in the West Midlands, United Kingdom. The study period covered all antenatal vitamin D testing performed between January 1, 2022, and December 31, 2022, allowing assessment of vitamin D status across a full calendar year.

Study population

During 2022, a total of 9,619 pregnant women booked for antenatal care at UHB. Among them, 2,757 had serum 25(OH)D levels measured at some point during pregnancy and were therefore eligible for inclusion. To enable detailed comparison between vitamin D categories, a random sample of 300 women was selected from this cohort, comprising 100 women with normal vitamin D levels, 100 with mild deficiency, and 100 with severe deficiency. Randomization ensured that the three groups were broadly comparable in terms of maternal age, parity, ethnicity, body mass index (BMI), and clinical risk factors.

Vitamin D measurement and classification

Serum 25(OH)D concentrations were measured by the hospital biochemistry laboratory using standardized NHS-validated assays. For the purpose of this study, vitamin D status was classified in line with UK Public Health and NICE criteria, which are also routinely used in clinical practice at UHB. Levels greater than 50 nmol/L were considered sufficient, concentrations between 25 and 49.9 nmol/L were classified as mild deficiency, and levels below 25 nmol/L were categorized as severe deficiency. These thresholds formed the basis for prevalence estimation and subgroup analyses.

Data collection

Data were retrieved from the hospital laboratory information system and the electronic maternity record. Variables collected included maternal age, ethnicity, parity, BMI, Index of Multiple Deprivation (IMD) quintile, breastfeeding and smoking status, and relevant comorbidities such as malabsorption syndromes, severe liver or kidney disease, and long-term steroid use. All identifiable information was removed prior to analysis, and data were handled in accordance with institutional data governance standards.

Outcome measures

The primary outcome of interest was the incidence and severity of vitamin D deficiency among pregnant women tested during the study period. Secondary outcomes included evaluating how deficiency was distributed across different demographic and clinical subgroups and assessing whether the current risk-based screening approach reliably identified women with low vitamin D levels.

Statistical analysis

All statistical analyses were performed using IBM SPSS Statistics (Version 26; IBM Corp., Armonk, NY, US). Descriptive statistics were generated for all variables, with categorical data presented as frequencies and percentages and continuous variables expressed as means with standard deviations or medians with interquartile ranges, depending on distribution. Group comparisons across the three vitamin D categories were conducted using the chi-squared test for categorical variables and one-way ANOVA or the Kruskal-Wallis test for continuous variables, as appropriate. A two-tailed p-value of <0.05 was considered statistically significant.

Ethical considerations

This study analyzed anonymized, routinely collected clinical and laboratory data. As it met criteria for service evaluation and did not involve direct patient recruitment or intervention, formal ethical approval was not required according to local NHS and institutional guidelines. All procedures adhered to the principles of patient confidentiality and Good Clinical Practice.

## Results

A total of 300 pregnant women with complete clinical records were included, comprising 100 women with normal vitamin D levels, 100 with mild deficiency, and 100 with severe deficiency. The mean maternal age was 29.94 ± 6.73 years, with ages ranging from 18 to 40 years. Age distribution showed that 76 women (25.3%) were between 18 and 25 years, 163 women (54.3%) were between 26 and 35 years, 44 women (14.7%) were between 36 and 40 years, and 17 women (5.7%) were above 40 years.

The mean BMI was 29.33 ± 7.72 kg/m², ranging from 15.0 to 60.2. BMI categories demonstrated that three women (1%) were underweight (BMI < 18), 106 women (35.3%) had normal BMI (18.1-25.9), 69 women (23%) were overweight (26-29.9), 110 women (36.7%) had class I-II obesity (30-40), and 12 women (4%) had morbid obesity (>40) (Table 1).

**Table 1:** Baseline Characteristics of the Study Sample (n = 300). BMI: body mass index

Characteristic	Frequency (n)	Percentage
Age (years)		
18–25	76	25.30%
26–35	163	54.30%
36–40	44	14.70%
>40	17	5.70%
BMI (kg/m²)		
<18	3	1.00%
18.1–25.9	106	35.30%
26–29.9	69	23.00%
30–40	110	36.70%
>40	12	4.00%
Vitamin D category		
Normal (>50 nmol/L)	100	33.30%
Mild deficiency (25–49.9 nmol/L)	100	33.30%
Severe deficiency (<25 nmol/L)	100	33.30%

Prevalence of vitamin D deficiency in the tested population

A total of 2,757 pregnant women had serum 25(OH)D levels recorded during the study period. Of these, 1,701 women (61.7%) demonstrated insufficient or deficient vitamin D levels (<50 nmol/L). A one-sample, one-tailed t-test was performed to determine whether the true population proportion of deficiency was not less than 60%. The result confirmed that the prevalence of vitamin D insufficiency/deficiency was significantly higher than the 60% threshold (p = 0.034), indicating a substantial burden of low vitamin D levels among pregnant women in our population.

Among the 1,701 women with low vitamin D levels, 634 women (37.3%) had severe deficiency (<25 nmol/L) (Table 2). To assess whether the proportion of severely deficient cases exceeded 21%, a one-sample, one-tailed t-test was conducted. The result showed that the true prevalence of severe deficiency was significantly higher than 21% (p = 0.006), highlighting a considerable level of clinically significant hypovitaminosis D in this population.

**Table 2:** Prevalence of Vitamin D Status Among Pregnant Women Tested (n = 2,757). One-sample, one-tailed t-tests assessed whether the observed prevalence of total vitamin D deficiency exceeded 60% and whether severe deficiency exceeded 21%. A p-value < 0.05 was considered significant.

Vitamin D category	Definition	Number of women (n)	Percentage	p-value
Normal	>50 nmol/L	1,056	38.30%	-
Mild deficiency	25–49.9 nmol/L	1,067	38.70%	-
Severe deficiency	<25 nmol/L	634	23.00%	0.006
Total insufficient/deficient	<50 nmol/L	1,701	61.70%	0.034

Comparison of risk factors across vitamin D categories

The distribution of potential risk factors-including maternal age, BMI, parity, ethnicity, deprivation index, and comorbidities-was compared across the three vitamin D categories using chi-squared analyses. Maternal age (χ²(6) = 6.08, p = 0.415), BMI category (χ²(8) = 14.81, p = 0.063), ethnicity (χ²(12) = 15.00, p = 0.242), breastfeeding status (χ²(2) = 0.98, p = 0.61), smoking status (χ²(2) = 1.42, p = 0.492), and comorbidities (χ²(12) = 10.71, p = 0.558) demonstrated no statistically significant association with vitamin D status. The deprivation index also showed no meaningful relationship with vitamin D levels (χ²(4) = 2.30, p = 0.690). In contrast, parity showed a statistically significant association with vitamin D category (χ²(6) = 20.63, p = 0.002). Overall, these findings suggest that low vitamin D levels were broadly distributed across demographic and clinical subgroups, with parity emerging as the only variable significantly associated with vitamin D status (Table 3).

**Table 3:** Comparison of Risk Factors Across Vitamin D Categories (n = 300). Chi-squared tests were used to compare the distribution of each risk factor across the three vitamin D status groups. A p-value < 0.05 was considered significant.

Risk factor	χ² (df)	p-value	Cramer’s V
Maternal age	6.08 (6)	0.415	0.1
Deprivation index	2.30 (4)	0.69	0.06
BMI category	14.81 (8)	0.063	0.16
Parity	20.63 (6)	0.002	0.19
Ethnicity	15.00 (12)	0.242	0.16
Breastfeeding status	0.98 (2)	0.61	0.06
Smoking status	1.42 (2)	0.49	0.07
Comorbidities	10.71 (12)	0.558	0.13

## Discussion

This study demonstrated a markedly high burden of vitamin D insufficiency and deficiency among pregnant women receiving antenatal care at our tertiary center in Birmingham. More than 61% of women tested had suboptimal vitamin D levels, and 23% had severe deficiency (<25 nmol/L). Statistical analyses confirmed that both overall deficiency and severe deficiency were significantly above expected thresholds, underscoring the magnitude of this public health concern in our population. These findings align with global estimates suggesting that over one billion individuals worldwide suffer from vitamin D deficiency, with pregnant women representing one of the most vulnerable groups [15].

The high prevalence observed in our population is biologically and clinically significant. Research indicates that 60%-80% of fetal 25(OH)D levels are directly derived from the mother [16], and to ensure that the fetus reaches a minimum serum level of 50 nmol/L, maternal concentrations must exceed approximately 75 nmol/L [17]. Low maternal vitamin D levels during pregnancy have been linked not only to well-established obstetric complications, such as pre-eclampsia, gestational diabetes, preterm birth, and impaired fetal bone development, but also to long-term health risks in the offspring, including increased susceptibility to asthma, type 1 diabetes, autism spectrum disorders, schizophrenia, and multiple sclerosis [18].

Deficiency is not uniformly distributed across populations, and our findings must be interpreted within the context of Birmingham’s unique demographic composition. Over the past several decades, there has been a substantial shift in the city’s ethnic distribution. According to the 2021 census, the White British population accounts for only 48.7%, while Asian communities represent 31%, Black communities 10.9%, mixed ethnic groups 4.8%, and other ethnicities 4.6% [19]. These groups-particularly South Asian, Middle Eastern, and Black populations-are known to have reduced cutaneous synthesis of vitamin D due to darker skin pigmentation and are therefore at higher risk of deficiency, especially in northern latitudes with limited ultraviolet exposure [20].

The global literature mirrors these patterns. Reported deficiency rates in pregnant women include 95.6% in Turkey [21], 89% in Pakistan [22], 80%-100% in China [23], and 70%-80% in India [24]. Conversely, the lowest rates have been documented in equatorial regions such as Nigeria, where combined deficiency and insufficiency was 39.4%, and deficiency alone was 29% [25]. These international figures reinforce that vitamin D deficiency is particularly severe in countries and regions with similar ethnic compositions and sunlight limitations to those observed in Birmingham.

One of the most important findings of our study is that none of the traditional risk factors-including age, BMI, ethnicity, deprivation index, smoking, or comorbidities-were significantly associated with vitamin D status in the 300-patient cohort. This suggests that deficiency is widespread and not restricted to the risk groups currently targeted by UK guidelines. This pattern implies that risk-based screening is unlikely to identify the majority of deficient women, supporting the need for a universal testing approach. Higher parity was significantly associated with lower vitamin D levels in our study. This relationship may reflect cumulative nutritional depletion, increased physiological demands across successive pregnancies, or reduced opportunities for sunlight exposure and supplementation adherence in women with multiple children. Similar findings have been reported in other populations, highlighting the need for targeted vitamin D screening and supplementation strategies for multiparous women [26].

The question of whether universal supplementation alone is sufficient remains debated. Although supplementation programs exist, evidence indicates they may be insufficient without baseline assessment [27]. For example, the United Kingdom’s “Healthy Start” program provides low-income pregnant women with a multivitamin containing 300 IU of vitamin D, yet studies have shown that routine supplementation without measuring serum levels does not significantly increase the proportion of women achieving sufficient vitamin D status [28]. Yamanouchi et al. [29] found no significant difference in vitamin D sufficiency between women who accepted vs. declined supplementation (p = 0.11), suggesting that unmeasured deficiency may persist despite supplementation.

International evidence similarly highlights that a one-size-fits-all supplementation strategy is inadequate. Mithal and Kalra [30] recommend 1,000-2,000 IU/day during the second and third trimesters for Asian populations, given their high deficiency rates and limited toxicity risk. This dose is substantially higher than the low-dose supplements provided in most standard antenatal multivitamins, again supporting the importance of measuring individual serum levels rather than assuming adequacy.

Taken together, the data from our study and global literature strongly indicate that vitamin D deficiency represents a significant and underrecognized public health challenge in pregnant women, particularly in ethnically diverse and northern metropolitan regions such as Birmingham. The combination of high prevalence, ethnic susceptibility, absence of discriminatory risk factors, and low cost of testing and treatment provides compelling evidence that universal screening at booking may be both justified and necessary. Such an approach may improve early detection, enable adequate dosing, and reduce the risk of preventable maternal and neonatal complications.

Although this study has limitations-chiefly its retrospective design and inability to correlate deficiency with pregnancy outcomes-it provides robust local evidence demonstrating the limitations of risk-based screening. Future prospective studies should evaluate whether universal vitamin D screening and appropriately tailored supplementation can improve maternal-fetal outcomes in high-risk populations.

## Conclusions

This study demonstrates a substantially high prevalence of vitamin D insufficiency and deficiency among pregnant women in our Birmingham antenatal population, with over 60% showing suboptimal levels and more than one-third exhibiting severe deficiency. Although most classical risk factors-including maternal age, BMI, ethnicity, deprivation status, and comorbidities-were not significantly associated with vitamin D status, parity emerged as the only variable showing a meaningful association, with multiparous women more likely to have lower vitamin D levels. This suggests that while deficiency is widespread across all demographic groups, women with higher parity may represent a particularly vulnerable subgroup requiring targeted attention. These findings highlight the limitations of current risk-based screening, which is unlikely to capture the majority of women with low vitamin D levels in our diverse, high-risk population. Given the low cost of testing, the high burden of deficiency, and the significant maternal and fetal health implications, universal vitamin D screening at booking may be a more effective strategy for early identification and timely treatment. Implementing such an approach-along with heightened vigilance for women with higher parity-may help reduce preventable complications associated with vitamin D deficiency and improve maternal and neonatal outcomes. Further prospective studies evaluating universal testing and targeted supplementation are warranted to strengthen the evidence base and inform regional and national practice guidelines.
